# Chinese Public Attitudes and Opinions on Health Policies During Public Health Emergencies: Sentiment and Topic Analysis

**DOI:** 10.2196/58518

**Published:** 2024-10-28

**Authors:** Min Liu, Shuo Yuan, Bingyan Li, Yuxi Zhang, Jia Liu, Cuixia Guan, Qingqing Chen, Jiayi Ruan, Lunfang Xie

**Affiliations:** 1 Department of Nursing The First Affiliated Hospital of University of Science and Technology of China Division of Life Sciences and Medicine, University of Science and Technology of China Hefei China; 2 Department of Cardiology II Anhui No.2 Provincial People's Hospital Hefei China; 3 The First Affiliated Hospital of Anhui Medical University Hefei China; 4 School of Nursing Anhui Medical University Hefei China

**Keywords:** public health emergencies, nucleic acid testing, governance strategies, sentiment analysis, LDA, social media, COVID-19, opinion analysis

## Abstract

**Background:**

By the end of 2021, the new wave of COVID-19 sparked by the Omicron variant spread rapidly due to its highly contagious nature, affecting more than 170 countries worldwide. Nucleic acid testing became the gold standard for diagnosing novel coronavirus infections. As of July 2022, numerous cities and regions in China have implemented regular nucleic acid testing policies, which have had a significant impact on socioeconomics and people’s lives. This policy has garnered widespread attention on social media platforms.

**Objective:**

This study took the newly issued regular nucleic acid testing policy during the COVID-19 pandemic as an example to explore the sentiment responses and fluctuations of netizens toward new policies during public health emergencies. It aimed to propose strategies for managing public opinion on the internet and provide recommendations for policy making and public opinion control.

**Methods:**

We collected blog posts related to nucleic acid testing on Weibo from April 1, 2022, to July 31, 2022. We used the topic modeling technique latent Dirichlet allocation (LDA) to identify the most common topics posted by users. We used Bidirectional Encoder Representations from Transformers (BERT) to calculate the sentiment score of each post. We used an autoregressive integrated moving average (ARIMA) model to examine the relationship between sentiment scores and changes over time. We compared the differences in sentiment scores across various topics, as well as the changes in sentiment before and after the announcement of the nucleic acid price reduction policy (May 22) and the lifting of the lockdown policy in Shanghai (June 1).

**Results:**

We collected a total of 463,566 Weibo posts, with an average of 3799.72 (SD 1296.06) posts published daily. The LDA topic extraction identified 8 topics, with the most numerous being the Shanghai outbreak, nucleic acid testing price, and transportation. The average sentiment score of the posts was 0.64 (SD 0.31), indicating a predominance of positive sentiment. For all topics, posts with positive sentiment consistently outnumbered those with negative sentiment (*χ*^2^_7_=24,844.4, *P*<.001). The sentiment scores of posts related to “nucleic acid testing price” decreased after May 22 compared with before (*t*_120_=3.882, *P*<.001). Similarly, the sentiment scores of posts related to the “Shanghai outbreak” decreased after June 1 compared with before (*t*_120_=11.943, *P*<.001).

**Conclusions:**

During public health emergencies, the topics of public concern were diverse. Public sentiment toward the regular nucleic acid testing policy was generally positive, but fluctuations occurred following the announcement of key policies. To understand the primary concerns of the public, the government needs to monitor social media posts by citizens. By promptly sharing information on media platforms and engaging in effective communication, the government can bridge the information gap between the public and government agencies, fostering a positive public opinion environment.

## Introduction

A public health emergency refers to an event that occurs suddenly and causes, or is likely to cause, serious harm to public health [1]. This includes major infectious disease outbreaks, mass unexplained illnesses, significant food and occupational poisoning, and other events that severely affect public health [1]. It is generally believed that public health emergencies are characterized by suddenness, group nature, diversity, high frequency, severe social harm, international interaction, and developmental stages [2]. With the acceleration of modernization, various uncertainties and unforeseeable risks continue to emerge, such as the COVID-19 pandemic and other public health emergencies [3]. Since the founding of the People’s Republic of China, the COVID-19 pandemic has represented the fastest-spreading, widest-ranging, and most challenging public health emergency in terms of prevention and control [4]. By the end of 2021, the new wave of COVID-19 sparked by the Omicron variant spread rapidly due to its highly contagious nature, affecting more than 170 countries worldwide. According to data released by the National Health Commission of China, from February 26 to May 4, 2022, there were a total of 54,886 locally confirmed cases of COVID-19 in China [5]. This number exceeded the total number of confirmed cases nationwide from the outbreak in Wuhan until the end of 2021 [5]. On October 19, 2022, the Director-General of the World Health Organization stated that the COVID-19 pandemic continues to constitute an “international public health emergency of concern” [6].

In responding to public health emergencies, government policies play an important role in controlling the pandemic and protecting public health [7]. The regular nucleic acid testing policy was a routine and periodic measure implemented by the Chinese government to control the spread of the epidemic [8]. The frequency of nucleic acid testing was as often as once a week or even more frequently [8-10] to achieve the objective of early detection, early reporting, early isolation, and early treatment in order to block the transmission of the virus as much as possible [10]. However, the effectiveness of these policies depends not only on their scientific and rational basis but also on the public’s acceptance and cooperation [11]. The regular nucleic acid testing had a significant impact on the economy, society, and people’s lives, sparking widespread public debate. In September 2022, the number of monthly active users of Sina Weibo reached 584 million, with a daily average of 253 million active users [12]. As one of the most widely used and influential social media platforms, Weibo has become a primary arena for users to obtain information about sudden events and express personal opinions [13]. By analyzing citizens’ views on the regular nucleic acid testing policy expressed on Weibo, the government can better understand public attitudes, evaluate and analyze policy effectiveness, and promptly adjust and optimize policy to enhance the scientific and effective management of emergencies [14].

Several studies used Weibo data to explore public sentiment changes during the COVID-19 pandemic, primarily investigating public opinions on the pandemic itself [15-17], COVID-19 vaccines [18-20], the Wuhan lockdown [21], social isolation measures [22], and rumors related to the pandemic [23,24]. However, there is a lack of research on public attitudes and opinions regarding the regular nucleic acid testing policy. Timely monitoring of public reactions to policies is crucial for the government to receive rapid feedback and make necessary policy adjustments during the pandemic [14]. Therefore, this study investigated the sentiment attitudes of the Chinese public during the first 3 months of the implementation of the regular nucleic acid testing policy and analyzed the changes in public attitudes over time and across different topics, as well as the reasons behind these changes. The expected results of this study could provide empirical evidence for policy makers to optimize policies and enhance public acceptance and implementation effectiveness, thereby contributing to the prevention and control of public opinion on social media. To our knowledge, this is the first study to explore the attitudes and opinions of the Chinese public toward regular nucleic acid testing during the COVID-19 pandemic, filling the gap in the existing literature on the attitudes of the Chinese public toward epidemic prevention policies during public health emergencies.

## Methods

### Data Collection and Preprocessing

**Figure 1:**
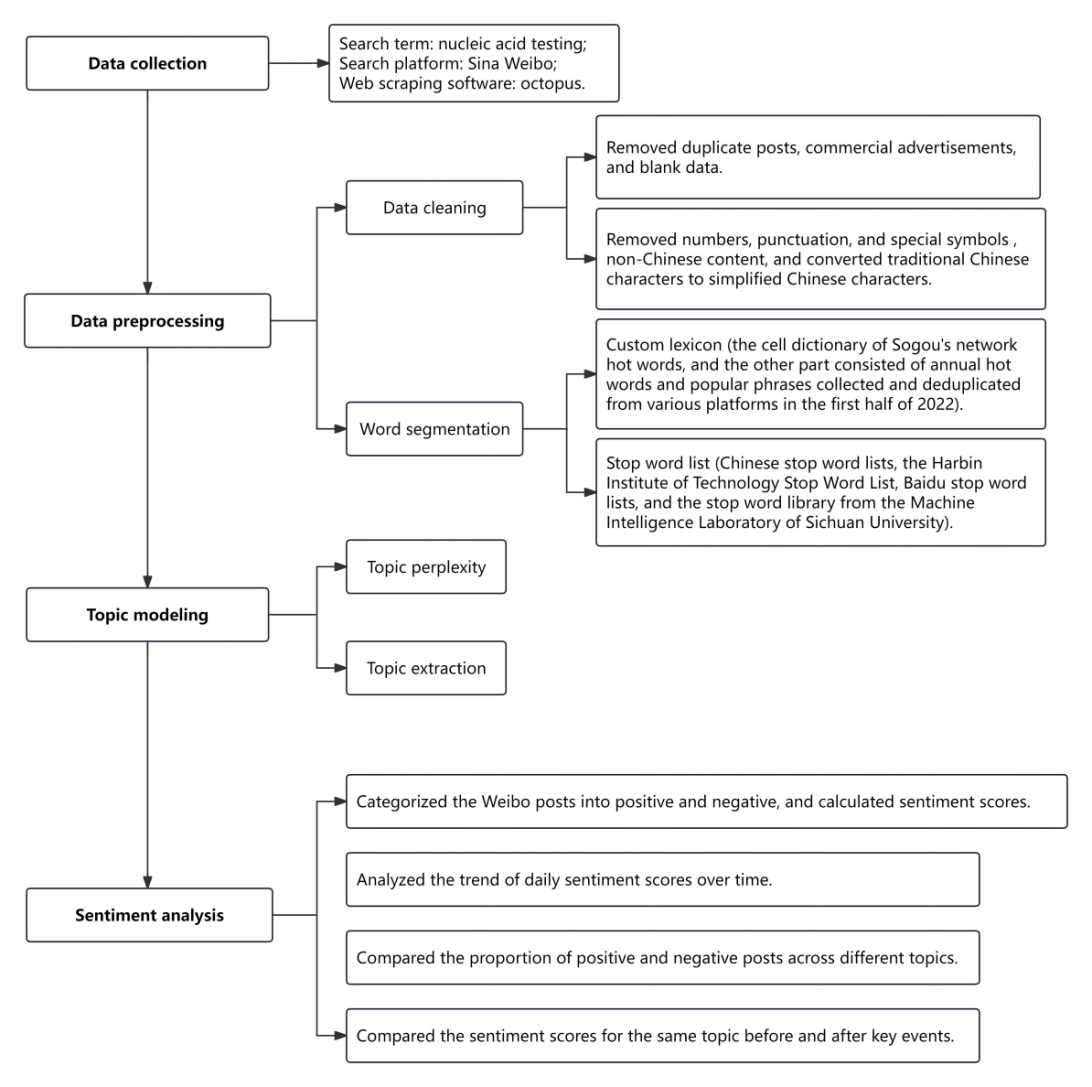
Data collection and processing workflow.

In April 2022, Shenzhen was the first city in China to set up regular nucleic acid testing sites and implement regular nucleic acid testing [25]. By July 2022, most regions across the country had conducted regular nucleic acid testing [26]. Therefore, we used the keyword “nucleic acid testing” to retrieve original Weibo data posted between April 1, 2022, and July 31, 2022. We used Octopus software (Shenzhen Skieer Information and Technology Co Ltd) to obtain data such as blog posts, posting times, and publisher nicknames. After removing duplicate posts, commercial advertisements, and blank data, a total of 463,566 Weibo posts were obtained. Afterward, data cleaning was performed, including removing numbers, punctuation, and special symbols (including URLs, @usernames, spaces, etc), removing non-Chinese content, and converting traditional Chinese characters to simplified Chinese characters. Tokenization was performed by using the Python Jieba package. Because many words on Weibo were internet neologisms and trending terms, and many were related to the COVID-19 pandemic, such as “health code,” “green code,” and even the main focus of this study, “nucleic acid testing,” might not have been correctly segmented. Therefore, we created a custom lexicon to assist with segmentation [27]. The custom lexicon was divided into 2 parts. One part was the cell dictionary of Sogou’s network hot words [28], and the other part consisted of annual hot words and popular phrases collected and deduplicated from various platforms in the first half of 2022, which were selected manually. The stop word list used included Chinese stop word lists, the Harbin Institute of Technology Stop Word List, Baidu stop word lists, and the stop word library from the Machine Intelligence Laboratory of Sichuan University [27]. More details are in Figure 1. At the same time, we obtained the daily number of newly confirmed cases from the official website of the Chinese Center for Disease Control and Prevention [29].

### Topic Modeling

This study used the Latent Dirichlet Allocation (LDA) model for topic modeling. LDA is the most widely used topic model at the time and could be used to identify the most common topics across social media platforms [30]. LDA is an unsupervised learning algorithm that does not require prelabeled datasets, making it suitable for handling large and diverse text data such as Weibo posts [31]. It is a Bayesian probabilistic model with 3 layers: “document-topic-word,” which is highly effective in identifying latent semantic topics in unstructured textual data, such as Weibo texts [32]. Furthermore, the LDA model does not have strict restrictions on the length of text; it is highly efficient in identifying topics in short texts [32]. Before constructing the LDA model, it is necessary to determine the number of topics [33]. Perplexity is a metric for determining whether the number of topics is reasonable, as the appropriate number of topics directly affects the accuracy of topic mining [33]. As the number of topic words increases, the perplexity value first decreases to a minimum point and then gradually increases [33]. The number of topic words when the perplexity reaches its lowest point is the optimal number of topics.

### Sentiment Analysis

We used a Bidirectional Encoder Representations from Transformers (BERT) to classify the sentiment of the data. The BERT uses a bidirectional transformer architecture, allowing it to understand each word in a sentence from both left to right and right to left simultaneously [34]. This bidirectionality enables BERT to capture the full contextual information, aiding in more accurately understanding complex sentence structures and sentiment expressions in Weibo posts [35]. In addition, Weibo often contains a large amount of unstructured text, emojis, acronyms, and other elements [36]. BERT, with its powerful language modeling and feature extraction capabilities, can effectively handle these characteristics, capturing subtle emotional changes and implicit sentiment information [34]. The model assigned a sentiment score between 0 and 1 to each Weibo post. As the sentiment score increased, positivity increased. Scores between 0 and 0.5 indicated negative sentiment, a score of 0.5 indicated neutrality, and scores between 0.5 and 1 indicated positive sentiment [37]. We tested the model’s accuracy and ensured it was above 85%.

### Statistical Analysis

All statistical analyses were conducted using IBM SPSS (version 24.0) and Python software (Python Software Foundation). We performed Spearman correlation analysis to explore the relationship between the daily number of posts with the number of newly confirmed cases per day. We used an autoregressive integrated moving average (ARIMA) model to examine the relationship between sentiment scores and changes over time. The chi-square test was used to compare the proportions of different types of sentiment posts under various topics. The *t* test was used to compare the sentiment scores at different times within the same topic. A 2-tailed test was conducted, and *P*<.05 was considered statistically significant.

### Ethical Considerations

The public information involved in this study is freely available on Sina Weibo without a password. All data are de-identified. In addition, there is no identifiable information about individual users, IDs, or non-paraphrased posts in the manuscript or any supplementary materials. No individuals were recruited as study subjects and therefore no ethical approval was required for this study.

## Results

### Number of Posts

**Figure 2:**
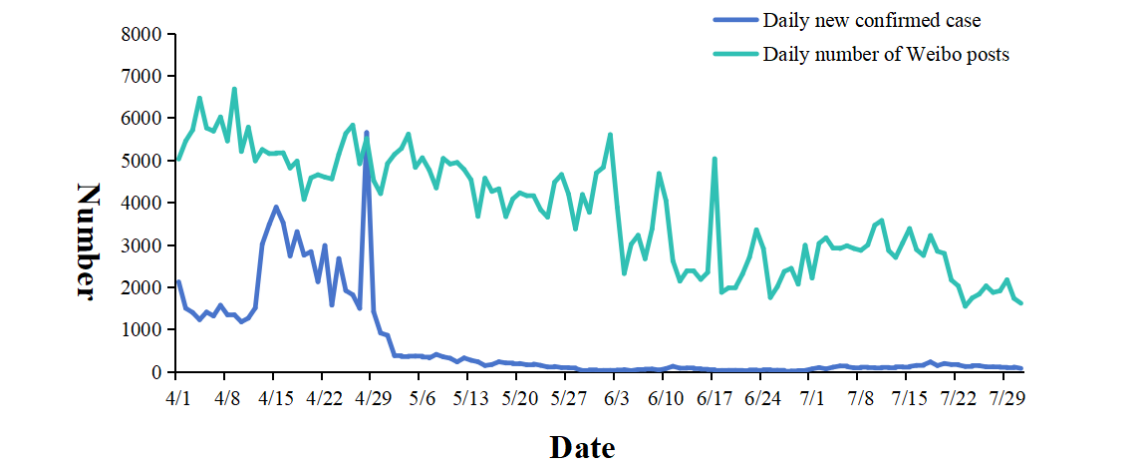
Daily changes in the number of nucleic acid tests and newly confirmed cases on Weibo.

From April to July 2022, there were a total of 463,566 posts about nucleic acid testing on Weibo, with an average of 3799.72 (SD 1296.06) posts per day. The highest number of posts was 6689 on April 9th, while the lowest number of posts was 1551 on July 23rd. Figure 2 shows the changes in the daily number of posts on Weibo and the daily number of newly confirmed cases from April to July, indicating a positive correlation between the two (ρ=0.656, *P*<.001).

### LDA Topic Extraction

#### Selection of Number of Topics

**Figure 3:**
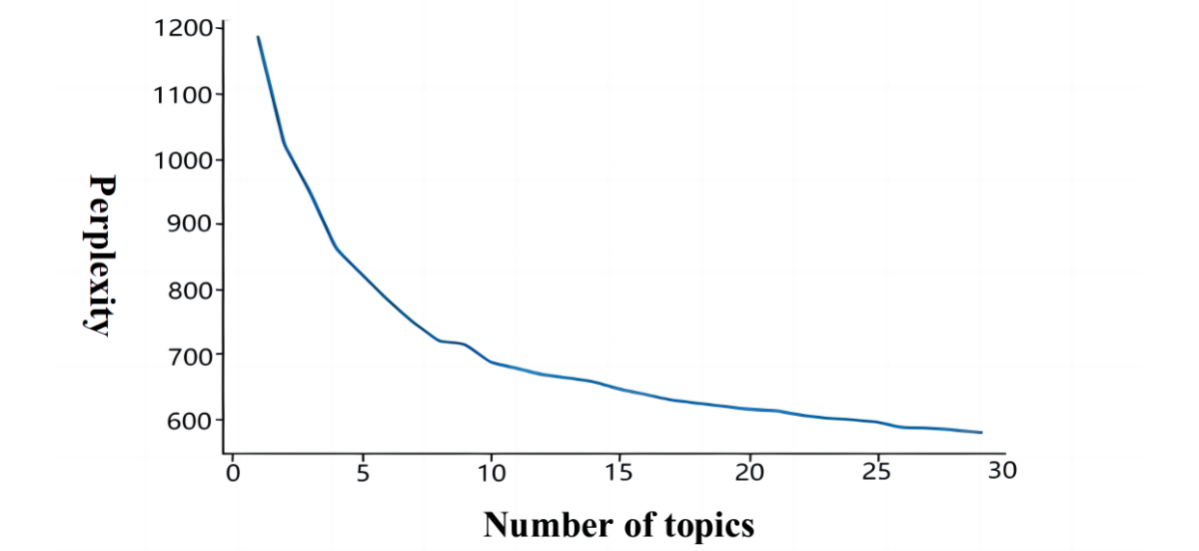
Perplexity of topics.

Figure 3 shows that the perplexity decreases as the number of topics in the LDA model increases. When the number of topics was K=8, the perplexity curve reached its lowest point and then rose before gradually decreasing again. Therefore, the number of topics selected in this study was K=8.

#### Topic Extraction

**Figure 4:**
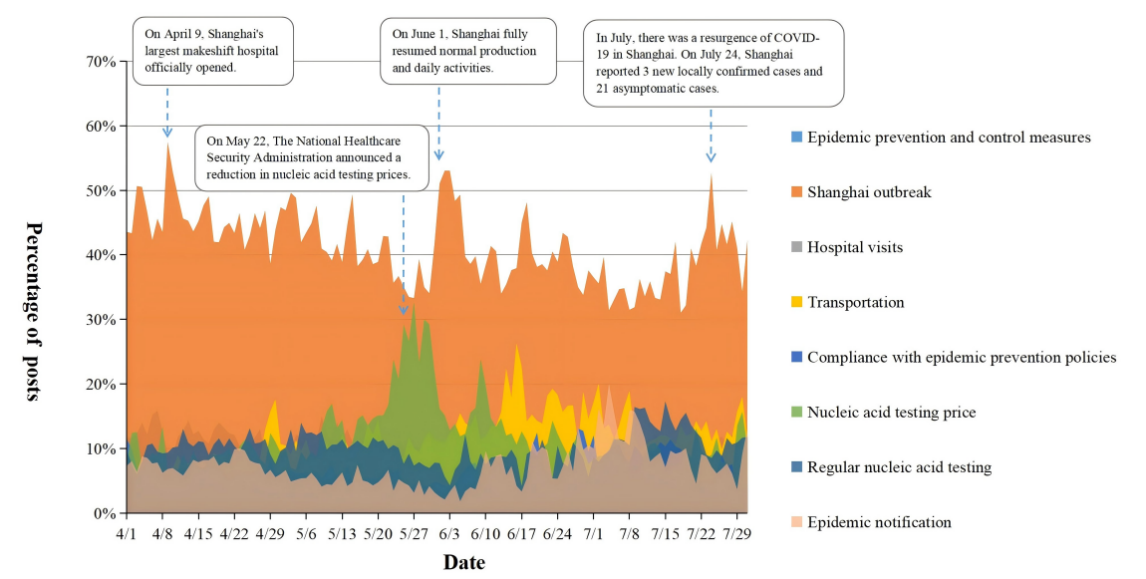
Changes in the number of posts on various topics over time.

Table 1 shows the 8 topics extracted by LDA and the proportion of posts on different topics. The top 10 high-frequency words were selected as the keywords for each topic, and based on these high-frequency keywords, the topic names were summarized according to the original Weibo texts [15]. In terms of quantity, the most frequently discussed topic was “Shanghai outbreak,” followed by “nucleic acid testing price” and “transportation.” The least discussed topic was “hospital visits.” Figure 4 describes the daily variation in the number of posts for each topic. From a temporal perspective, posts about the “Shanghai outbreak” peaked on April 9, while posts about “nucleic acid testing price” peaked on May 27.

**Table 1:** Latent Dirichlet allocation topic classification of Weibo nucleic acid testing–related posts.

Topic	Posts, n (%^a^)	Keywords
Epidemic prevention and control measures	48,284 (10.42)	Work, Epidemic Prevention and Control, Community, Volunteers, Service, Guarantee, Residents, Doing Well, Masses, Supplies
Shanghai outbreak	195,612 (42.20)	Community, Shanghai, Nucleic Acid Testing, Video, Queuing, Epidemic, Nucleic Acid Testing Site, Tomorrow, Time, Hope
Hospital visits	2137 (0.46)	Hospital, Opening, Sampling Point, Waiting, Number of People, Fever Clinic, Tianjin, People’s, Affiliated, Central Hospital
Transportation	51,101 (11.02)	Hours, Negative, Certificate, Health Code, Verification, Vehicle, Highway, Green Code, Travel Code, Travel
Compliance with epidemic prevention policies	31,033 (6.69)	Implementation, Measures, Masks, Cooperation, Community, Proactively, Reporting, Risk, Concentrated Isolation, Control
Nucleic acid testing price	55,502 (11.97)	Nucleic Acid Testing, Institutions, News, Beijing, Shanghai, Price, National, Laboratory, Press Conference, Normalized Nucleic Acid
Regular nucleic acid testing	47,157 (10.17)	Sampling, Area, Command Center, Notice, Community, Participation, Regular Nucleic Acid Testing, Staff, Cooperation, Arrangement
Epidemic notification	32,740 (7.06)	COVID-19 positive, Asymptomatic Carrier, Confirmed Cases, New Cases, Infected Individuals, Local Cases, Isolation, Diagnosis, Nucleic Acid Test Results, Report

^a^The percentages have been rounded to 2 decimal places for presentation clarity, leading to a total of 99.99%.

### Sentiment Analysis

#### The Temporal Trend in Sentiment Score

**Figure 5:**
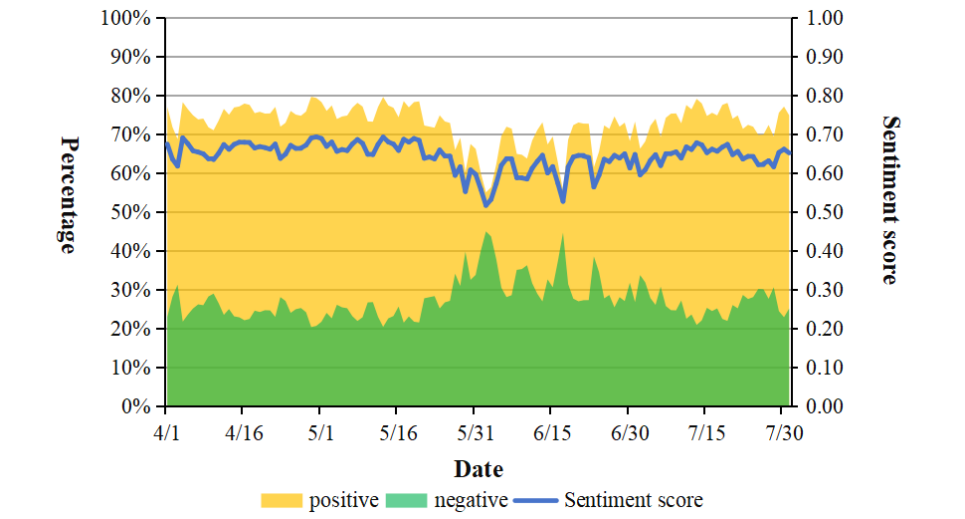
The dynamic changes in daily sentiment scores, the number of positive posts, and the number of negative posts.

In terms of sentiment polarity scores, the average sentiment score for posts about nucleic acid testing was 0.64 (SD 0.31), with daily sentiment scores fluctuating between 0.52 and 0.69. Since there were no posts with neutral sentiment, the posts in this study included only positive and negative sentiment. Among them, there were 337,968 posts with positive sentiment, accounting for 72.91% (337,968/463,566), and 125,598 posts with negative sentiment, accounting for 27.09% (125,598/463,566). Figure 5 shows the temporal changes in sentiment polarity of posts from April to July, with sentiment scores hitting low points on June 2 and June 17.

**Figure 6:**
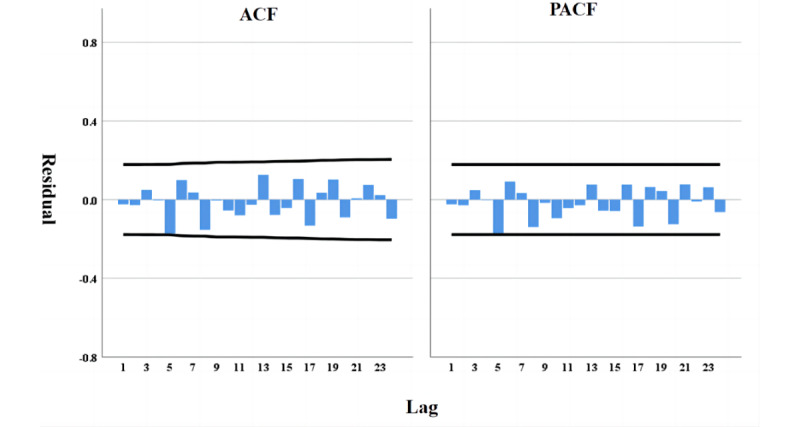
Residual ACF and PACF. ACF: auto-correlation function. PACF：partial auto-correlation function.

**Figure 7:**
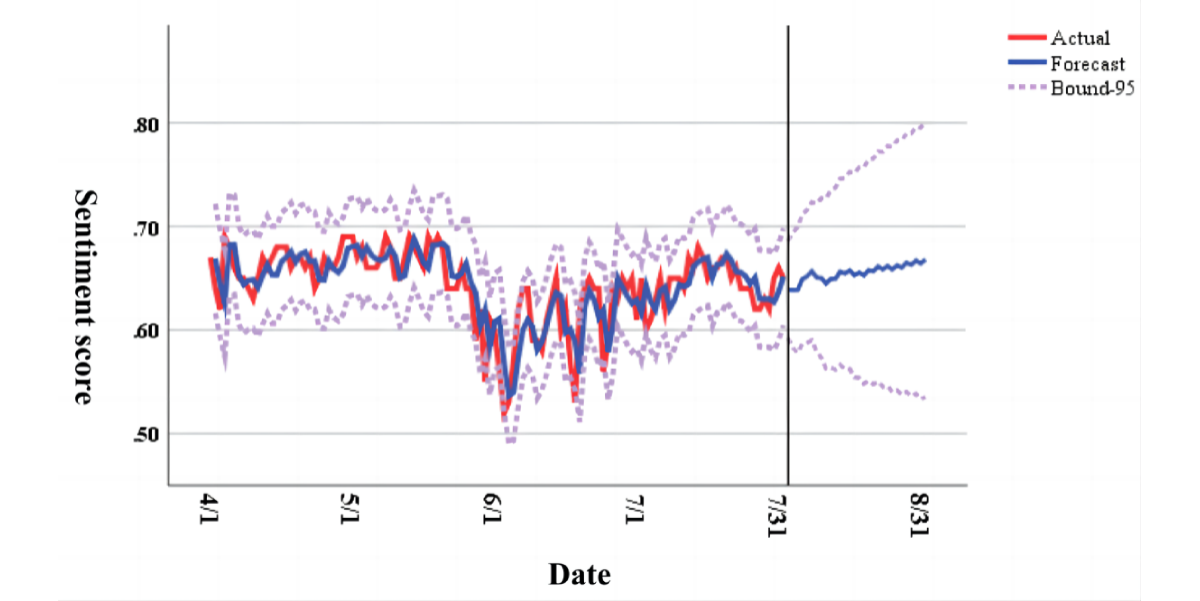
A time series plot of daily sentiment score from April 2022 to August 2022 predicted using the ARIMA model.

We conducted a stationarity test on the original time series and found it to be nonstationary. We performed first-order differencing and used auto-correlation function (ACF) and partial auto-correlation function (PACF) plots to determine that d=1, p=4, and q=3. Subsequently, we conducted a white noise test on the residuals. The auto-correlation function plot of the results showed no significant auto-correlation, indicating no temporal auto-correlation, as shown in Figure 6. We used the ARIMA (4, 1, 3) model for our analysis, with the model fitting metrics provided in Multimedia Appendix 1. Based on the model, we predicted the sentiment trend for August 2022. As shown in Figure 7, there were some fluctuations in Weibo sentiment in August, but overall, positive sentiment remained dominant.

#### Sentiment Scores for Different Topics

Table 2 shows the emotional scores across different topics. We found that all topics have positive sentiment scores, with the proportion of positive posts higher than negative ones (*χ*^2^_7_=24844.4, *P*<.001). Specifically, the topics “Shanghai outbreak” and “nucleic acid testing price” had over 30% negative sentiment posts. The daily sentiment score heat maps for different topics are shown in Multimedia Appendix 2.

**Table 2:** Sentiment scores for 8 topics and a comparison of positive and negative posts across different topics.

Topic	Sentiment score	Proportion of positive posts, n (%)	Proportion of negative posts, n (%)	Chi-square (*df*)	*P* value
Epidemic prevention and control measures	0.74	41,074 (85.07)	7210 (14.93)	24,844.4 (7)	<.001
Shanghai outbreak	0.59	124,009 (63.40)	71,603 (36.60)	—^a^	—
Hospital visits	0.64	1710 (80.02)	427 (19.98)	—	—
Transportation	0.63	37,247 (72.89)	13,854 (27.11)	—	—
Compliance with epidemic prevention policies	0.77	28,532 (91.94)	2501 (8.06)	—	—
Nucleic acid testing price	0.62	38,123 (68.69)	17,379 (31.31)	—	—
Regular nucleic acid testing	0.72	41,524 (88.05)	5633 (11.95)	—	—
Epidemic notification	0.68	25,749 (78.65)	6991 (21.35)	—	—

^a^Not applicable.

#### Sentiment Changes Before and After Key Time Points

On May 22, 2022, the National Healthcare Security Administration announced a reduction in nucleic acid testing prices [38]. Shanghai announced on June 1, 2022, the full restoration of production order [39]. As shown in Table 3, both the topics of “nucleic acid testing price” and the “Shanghai outbreak” maintained a positive attitude during different time periods. Before May 22, the sentiment scores of posts related to “nucleic acid testing prices” were higher than they were after May 22 (*t*_120_=3.882, *P*<.001). Before June 1, the sentiment scores of posts about the “Shanghai outbreak” were higher than they were after June 1 (*t*_120_=11.943, *P*<.001).

**Table 3:** Changes in sentiment scores across different time periods.

Topic			Sentiment score, Mean (SD)		*t* test (*df*)		*P* value
**Nucleic acid testing price**			—^a^		3.882 (120)		<.001
	Before May 22	0.66 (0.06)		—		—	
	After May 22	0.61 (0.07)		—		—	
**Shanghai outbreak**			—		11.943 (120)		<.001
	Before June 1	0.61 (0.03)		—		—	
	After June 1	0.55 (0.03)		—		—	

^a^Not applicable.

## Discussion

### Principal Findings

This paper explored public attitudes and opinions on regular nucleic acid testing on Weibo using sentiment and topic analysis. It analyzed public sentiment expressions on social media in the context of public health emergencies. To our knowledge, this is the first study to explore public attitudes and opinions on regular nucleic acid testing during the COVID-19 pandemic, filling a gap in existing research. The results of this study can help the government promptly understand changes in public sentiment during future emergencies, effectively guide public emotions, manage negative emotions, and maintain social order.

### Positive Correlation Between the Volume of Weibo Posts and the Severity of the Epidemic

The study results showed a positive correlation between the daily number of posts on the topic of regular nucleic acid testing on Chinese social media and the number of new confirmed COVID-19 cases in China (ρ=0.656, *P*<.001). As the number of new confirmed cases increased, the government increased the frequency of nucleic acid testing to identify and control the outbreak more quickly. This led to more people participating in testing and sharing their experiences and views on social media. Our findings were consistent with a study on epidemic-related rumors on Weibo from January 20 to May 28, 2020, which found a positive correlation between the daily number of posts about COVID-19 misinformation on Chinese social media and the daily number of newly confirmed and newly suspected COVID-19 cases in China [40]. This indicated that the dissemination of information about COVID-19 in China was synchronized with the spread of the epidemic [40]. The volume of discussions on social media can serve as a supplementary indicator for predicting epidemic trends [41]. By monitoring public opinion on social media in real time, public health departments can promptly understand public concerns and provide early warnings for emerging outbreaks, thereby better formulating and adjusting control measures [41].

### The 3 Topics of Primary Concern to the Public

This study identified 8 main topics of public interest regarding regular nucleic acid testing. Ranked in order of attention from highest to lowest, they were the Shanghai outbreak, nucleic acid testing price, transportation, epidemic prevention and control measures, regular nucleic acid testing, epidemic notification, and hospital visits. Wang used the Weibo platform to investigate public concerns during the early stage of the pandemic (December 1, 2019, to July 31, 2020), identifying 8 topics, including epidemic notification, prevention measures, and health care [15]. In comparison, this study found that after the implementation of the regular nucleic acid testing policy, public attention shifted more toward topics such as the Shanghai outbreak, nucleic acid testing price, and transportation. Analyzing the reasons, the period focused on in this study coincided with the outbreak of the pandemic in Shanghai, during which multiple rounds of large-scale mass nucleic acid testing were conducted [42]. The frequent testing led to public concerns about the costs of nucleic acid testing, and any government policy adjustments regarding these costs attracted significant attention. Simultaneously, as many regions required travelers to provide proof of a negative nucleic acid test, public transportation and travel faced strict restrictions. Therefore, the Shanghai outbreak, nucleic acid testing price, and transportation have become focal points of public concern due to their close relation to public health and daily life. The government can determine the priority of public concerns based on the volume of posts on various topics on social media and promptly disclose relevant policies and information on epidemic prevention and control.

### Public’s Positive Emotion at the Regular Nucleic Acid Testing Policy

Sentiment analysis results showed that public emotions toward regular nucleic acid testing were predominantly positive, though there were periods of downturn influenced by certain events. The positive sentiment in Weibo posts indicated that the public generally approved of the regular nucleic acid testing policy, which is consistent with the conclusions of the study by Tan [43]. From June 2021 to August 2021, Tan [43] analyzed the opinions of residents in 4 cities in Guangdong Province on nucleic acid testing using semistructured interviews and social media comments. The results revealed that the public generally held a positive attitude toward nucleic acid testing [43]. Other studies have shown that sudden policy changes provoke heated discussions among the public on social media platforms [14]. If timely explanations and public communication are not provided, this can lead to public concern and anxiety, resulting in a surge of negative emotions [14]. On June 1, the Shanghai government suddenly announced adjustments to its pandemic prevention policies, leading to an increase in negative public emotions [39]. By June 2, the sentiment score had reached its lowest point. Fortunately, public negative emotions decreased afterward, and sentiment gradually stabilized. Support for regular nucleic acid testing increased over time. Decision makers should closely monitor changes in public reactions to address the most critical public needs at different stages of the pandemic [14]. By doing so, they can address public concerns through timely and effective communication [14]. In addition, policy makers can leverage social media and big data analysis to support public health decision-making [44].

### Impact of Price Reduction Policy on Sentiment Scores of “Nucleic Acid Testing Price”

Due to the economic burden on the public induced by regular nucleic acid testing, the government adjusted the prices of nucleic acid tests multiple times. On May 22, 2022, the National Healthcare Security Administration issued a notice to reduce the prices of nucleic acid testing [38]. However, after the policy was implemented, the sentiment score in Weibo posts, while still positive, was lower than before the policy was issued. Analyzing the reasons, first, although the cost of regular nucleic acid testing was primarily subsidized by the government, the requirement to undergo testing every few days disrupted the public’s daily life [45]. Second, when the price reduction policy was first announced, media reports sometimes highlighted inconsistencies between local and central policies or issues with policy implementation. Such negative reports could raise public doubts and increase the proportion of negative emotions. Studies have shown that after the introduction of new policies, insufficient government explanation or failures in public communication can greatly affect public perception and trust due to misinformation [46]. Therefore, when introducing new policies, the government should release information in a timely and transparent manner, including specific implementation details and progress, to reduce information asymmetry. During the policy implementation process, the government should establish diverse channels for public feedback, gather public opinions and suggestions, and promptly respond to and address public concerns to enhance the effectiveness of policy implementation.

### Impact of Reopening Policy on Sentiment Scores of “Shanghai Outbreak”

Among all topics, the proportion of negative emotions regarding the “Shanghai outbreak” reached 36.60%, and the public sentiment score declined after June 1. Since the outbreak of the pandemic in March 2022, the number of infections in Shanghai had grown exponentially [47]. On April 1, the Shanghai government decided to implement city-wide static management, initiating a lockdown that lasted for 3 months [42]. Studies have shown that quarantine can lead to negative psychological effects such as posttraumatic stress symptoms, confusion, and anger [21,22]. On June 1, Shanghai fully resumed normal life [39]. However, during the actual process of resuming work and production, various issues were encountered, leading to strong negative emotions expressed by people on Weibo, which caused a decline in public sentiment scores. Furthermore, although the restoration of production and daily life was underway, the risk of a resurgence of the pandemic remained. This caused public concern about the future development of the pandemic, leading to a decline in sentiment scores. In addition, numerous rumors about the Shanghai outbreak circulated on the internet, contributing to negative emotions [23]. Previous research has found that public sentiment typically shifts from negative to positive after rumors are debunked [24]. Guo investigated public attitudes toward the Shanghai outbreak on the Weibo platform and found that public sentiment scores consistently declined during the outbreak, falling into negative territory in the second and third quarters [48]. The severe negative emotions among the public significantly affected Shanghai’s image [48]. Therefore, after announcing policies, the government needs to promptly track trends in public sentiment, identify potential negative emotions and hot issues, and respond effectively to control negative emotions within reasonable limits. In addition, the government should proactively guide public opinion by using authoritative media and expert interpretations to effectively disseminate policies related to public health emergencies, thereby reducing the spread of rumors. Furthermore, the public needs to enhance their ability to identify and not believe or spread rumors. Joint efforts from the government, media platforms, and the public are essential to create a positive and uplifting public opinion environment.

### Limitations

This study has several limitations. First, the Weibo data collected used only 1 keyword, which may include only a portion of posts related to nucleic acid testing rather than all relevant posts. Therefore, the summary of the main emotions and topics of Weibo users may not be comprehensive. Second, most Weibo users are young people, so the analysis results may be more biased toward the perspectives of young people [49]. Third, we collected data from only 1 social media platform. Although Weibo has a relatively large user base in China, it is hasty to conclude that the opinions expressed on Weibo represent the views of the public [50]. Finally, due to the limitations of the Weibo platform and its functionalities, most information regarding user geographical location and other details collected in this study was missing [51]. Consequently, the study did not explore whether users from different geographic locations had different sentiments and topics of concern regarding the regular nucleic acid testing policy.

### Conclusions

During public health emergencies, the topics of public concern were multifaceted. Public sentiment toward regular nucleic acid testing policies was generally positive, but fluctuations occurred following key policy announcements. The government needs to monitor information posted by the public on social media platforms to understand the main areas of concern. By promptly disclosing information on media platforms and engaging in effective communication, the government can bridge the information gap between public needs and government responses to create a favorable public opinion environment.

## Appendix

Multimedia Appendix 1 Autoregressive Integrated Moving Average (ARIMA) model fit metrics and ARIMA model predicted daily sentiment scores.

Multimedia Appendix 2 Daily sentiment score heatmaps for different topics.

## Abbreviations

ACF: auto-correlation function
ARIMA: Autoregressive Integrated Moving Average
BERT: Bidirectional Encoder Representations from Transformers
LDA: latent Dirichlet allocation
PACF: partial auto-correlation function
