# Supplementary material for: Chinese Public Attitudes and Opinions on Health Policies During Public Health Emergencies: Sentiment and Topic Analysis
Source: J Med Internet Res. 2024 Oct 28;26:e58518. doi: 10.2196/58518 (PMC11555446; doi:10.2196/58518)
Supplement: Multimedia Appendix 1 [file jmir_v26i1e58518_app1.doc]

|  | *R*^2^ | RMSE | MAPE | MAE |
| --- | --- | --- | --- | --- |
| ARIMA (4,1,3) | 0.583 | 0.024 | 2.792 | 0.017 |

Table A1. ARIMA model fitting metrics.

Table A2. ARIMA Model Predicted Daily Sentiment Scores (April-August 2022).

| Date | Actual Sentiment Scores | Predicted Sentiment Scores (95% CI) |
| --- | --- | --- |
| 4/1 | 0.67 | —— |
| 4/2 | 0.64 | 0.67(0.62-0.72) |
| 4/3 | 0.62 | 0.65(0.59-0.7) |
| 4/4 | 0.69 | 0.63(0.57-0.68) |
| 4/5 | 0.68 | 0.68(0.63-0.73) |
| 4/6 | 0.66 | 0.68(0.63-0.73) |
| 4/7 | 0.65 | 0.65(0.61-0.7) |
| 4/8 | 0.65 | 0.64(0.6-0.69) |
| 4/9 | 0.64 | 0.65(0.6-0.7) |
| 4/10 | 0.63 | 0.65(0.6-0.7) |
| 4/11 | 0.65 | 0.64(0.59-0.69) |
| 4/12 | 0.67 | 0.65(0.61-0.7) |
| 4/13 | 0.66 | 0.66(0.62-0.71) |
| 4/14 | 0.67 | 0.65(0.61-0.7) |
| 4/15 | 0.68 | 0.65(0.61-0.7) |
| 4/16 | 0.68 | 0.67(0.62-0.71) |
| 4/17 | 0.68 | 0.67(0.62-0.72) |
| 4/18 | 0.66 | 0.68(0.63-0.72) |
| 4/19 | 0.67 | 0.67(0.62-0.71) |
| 4/20 | 0.67 | 0.67(0.63-0.72) |
| 4/21 | 0.66 | 0.68(0.63-0.72) |
| 4/22 | 0.67 | 0.67(0.62-0.71) |
| 4/23 | 0.64 | 0.67(0.62-0.71) |
| 4/24 | 0.65 | 0.65(0.6-0.7) |
| 4/25 | 0.67 | 0.65(0.6-0.7) |
| 4/26 | 0.66 | 0.67(0.62-0.71) |
| 4/27 | 0.66 | 0.66(0.61-0.71) |
| 4/28 | 0.67 | 0.66(0.61-0.7) |
| 4/29 | 0.69 | 0.66(0.61-0.71) |
| 4/30 | 0.69 | 0.68(0.63-0.73) |
| 5/1 | 0.69 | 0.68(0.63-0.73) |
| 5/2 | 0.67 | 0.68(0.63-0.73) |
| 5/3 | 0.68 | 0.67(0.62-0.72) |
| 5/4 | 0.66 | 0.68(0.63-0.73) |
| 5/5 | 0.66 | 0.67(0.63-0.72) |
| 5/6 | 0.66 | 0.67(0.62-0.72) |
| 5/7 | 0.67 | 0.67(0.62-0.71) |
| 5/8 | 0.69 | 0.67(0.62-0.72) |
| 5/9 | 0.68 | 0.68(0.63-0.73) |
| 5/10 | 0.65 | 0.67(0.62-0.72) |
| 5/11 | 0.65 | 0.65(0.6-0.7) |
| 5/12 | 0.67 | 0.65(0.61-0.7) |
| 5/13 | 0.69 | 0.67(0.63-0.72) |
| 5/14 | 0.68 | 0.69(0.64-0.73) |
| 5/15 | 0.67 | 0.68(0.63-0.72) |
| 5/16 | 0.66 | 0.66(0.62-0.71) |
| 5/17 | 0.69 | 0.66(0.61-0.71) |
| 5/18 | 0.68 | 0.68(0.64-0.73) |
| 5/19 | 0.69 | 0.68(0.64-0.73) |
| 5/20 | 0.68 | 0.68(0.64-0.73) |
| 5/21 | 0.64 | 0.68(0.63-0.73) |
| 5/22 | 0.64 | 0.65(0.61-0.7) |
| 5/23 | 0.64 | 0.65(0.6-0.7) |
| 5/24 | 0.66 | 0.65(0.61-0.7) |
| 5/25 | 0.64 | 0.66(0.62-0.71) |
| 5/26 | 0.64 | 0.65(0.6-0.69) |
| 5/27 | 0.59 | 0.64(0.59-0.68) |
| 5/28 | 0.62 | 0.6(0.56-0.65) |
| 5/29 | 0.55 | 0.62(0.57-0.67) |
| 5/30 | 0.61 | 0.58(0.54-0.63) |
| 5/31 | 0.60 | 0.61(0.56-0.65) |
| 6/1 | 0.56 | 0.61(0.56-0.66) |
| 6/2 | 0.52 | 0.57(0.52-0.61) |
| 6/3 | 0.53 | 0.54(0.49-0.58) |
| 6/4 | 0.57 | 0.54(0.49-0.59) |
| 6/5 | 0.62 | 0.57(0.53-0.62) |
| 6/6 | 0.64 | 0.6(0.55-0.65) |
| 6/7 | 0.64 | 0.61(0.56-0.66) |
| 6/8 | 0.59 | 0.6(0.56-0.65) |
| 6/9 | 0.59 | 0.58(0.53-0.63) |
| 6/10 | 0.58 | 0.59(0.54-0.64) |
| 6/11 | 0.61 | 0.6(0.56-0.65) |
| 6/12 | 0.63 | 0.62(0.58-0.67) |
| 6/13 | 0.65 | 0.64(0.59-0.68) |
| 6/14 | 0.60 | 0.63(0.59-0.68) |
| 6/15 | 0.62 | 0.6(0.55-0.64) |
| 6/16 | 0.57 | 0.6(0.55-0.65) |
| 6/17 | 0.53 | 0.59(0.54-0.64) |
| 6/18 | 0.62 | 0.56(0.51-0.6) |
| 6/19 | 0.64 | 0.62(0.58-0.67) |
| 6/20 | 0.65 | 0.64(0.59-0.68) |
| 6/21 | 0.64 | 0.63(0.58-0.68) |
| 6/22 | 0.64 | 0.61(0.56-0.66) |
| 6/23 | 0.56 | 0.62(0.57-0.67) |
| 6/24 | 0.59 | 0.58(0.53-0.63) |
| 6/25 | 0.64 | 0.61(0.56-0.65) |
| 6/26 | 0.63 | 0.65(0.6-0.7) |
| 6/27 | 0.65 | 0.64(0.59-0.69) |
| 6/28 | 0.64 | 0.63(0.59-0.68) |
| 6/29 | 0.65 | 0.63(0.58-0.67) |
| 6/30 | 0.61 | 0.63(0.58-0.68) |
| 7/1 | 0.65 | 0.62(0.57-0.66) |
| 7/2 | 0.60 | 0.64(0.6-0.69) |
| 7/3 | 0.61 | 0.63(0.58-0.67) |
| 7/4 | 0.63 | 0.62(0.57-0.67) |
| 7/5 | 0.65 | 0.64(0.59-0.68) |
| 7/6 | 0.62 | 0.64(0.59-0.69) |
| 7/7 | 0.65 | 0.62(0.57-0.67) |
| 7/8 | 0.65 | 0.63(0.58-0.68) |
| 7/9 | 0.65 | 0.64(0.6-0.69) |
| 7/10 | 0.64 | 0.64(0.59-0.69) |
| 7/11 | 0.67 | 0.64(0.6-0.69) |
| 7/12 | 0.66 | 0.66(0.61-0.71) |
| 7/13 | 0.68 | 0.67(0.62-0.71) |
| 7/14 | 0.67 | 0.67(0.62-0.72) |
| 7/15 | 0.65 | 0.67(0.62-0.72) |
| 7/16 | 0.66 | 0.65(0.6-0.7) |
| 7/17 | 0.66 | 0.66(0.62-0.71) |
| 7/18 | 0.67 | 0.66(0.62-0.71) |
| 7/19 | 0.67 | 0.67(0.63-0.72) |
| 7/20 | 0.65 | 0.67(0.62-0.71) |
| 7/21 | 0.66 | 0.66(0.61-0.7) |
| 7/22 | 0.64 | 0.66(0.61-0.7) |
| 7/23 | 0.64 | 0.65(0.61-0.7) |
| 7/24 | 0.64 | 0.64(0.6-0.69) |
| 7/25 | 0.62 | 0.65(0.6-0.7) |
| 7/26 | 0.62 | 0.63(0.58-0.68) |
| 7/27 | 0.63 | 0.63(0.58-0.68) |
| 7/28 | 0.62 | 0.63(0.58-0.68) |
| 7/29 | 0.65 | 0.63(0.58-0.67) |
| 7/30 | 0.66 | 0.64(0.59-0.68) |
| 7/31 | 0.65 | 0.65(0.6-0.7) |
| 8/1 | —— | 0.64(0.59-0.69) |
| 8/2 | —— | 0.64(0.58-0.69) |
| 8/3 | —— | 0.64(0.58-0.7) |
| 8/4 | —— | 0.65(0.59-0.71) |
| 8/5 | —— | 0.65(0.59-0.72) |
| 8/6 | —— | 0.66(0.59-0.72) |
| 8/7 | —— | 0.65(0.58-0.72) |
| 8/8 | —— | 0.65(0.57-0.73) |
| 8/9 | —— | 0.64(0.56-0.73) |
| 8/10 | —— | 0.65(0.56-0.73) |
| 8/11 | —— | 0.65(0.56-0.74) |
| 8/12 | —— | 0.66(0.57-0.75) |
| 8/13 | —— | 0.65(0.56-0.75) |
| 8/14 | —— | 0.66(0.56-0.75) |
| 8/15 | —— | 0.65(0.55-0.75) |
| 8/16 | —— | 0.66(0.55-0.76) |
| 8/17 | —— | 0.65(0.55-0.76) |
| 8/18 | —— | 0.66(0.55-0.76) |
| 8/19 | —— | 0.66(0.55-0.77) |
| 8/20 | —— | 0.66(0.55-0.77) |
| 8/21 | —— | 0.66(0.54-0.77) |
| 8/22 | —— | 0.66(0.55-0.78) |
| 8/23 | —— | 0.66(0.54-0.78) |
| 8/24 | —— | 0.66(0.54-0.78) |
| 8/25 | —— | 0.66(0.54-0.78) |
| 8/26 | —— | 0.66(0.54-0.79) |
| 8/27 | —— | 0.66(0.54-0.79) |
| 8/28 | —— | 0.67(0.54-0.8) |
| 8/29 | —— | 0.66(0.53-0.79) |
| 8/30 | —— | 0.67(0.53-0.8) |
| 8/31 | —— | 0.67(0.53-0.8) |
