# Supplementary figures and images for: Chinese Public Attitudes and Opinions on Health Policies During Public Health Emergencies: Sentiment and Topic Analysis
Source: J Med Internet Res. 2024 Oct 28;26:e58518. doi: 10.2196/58518 (PMC11555446; doi:10.2196/58518)

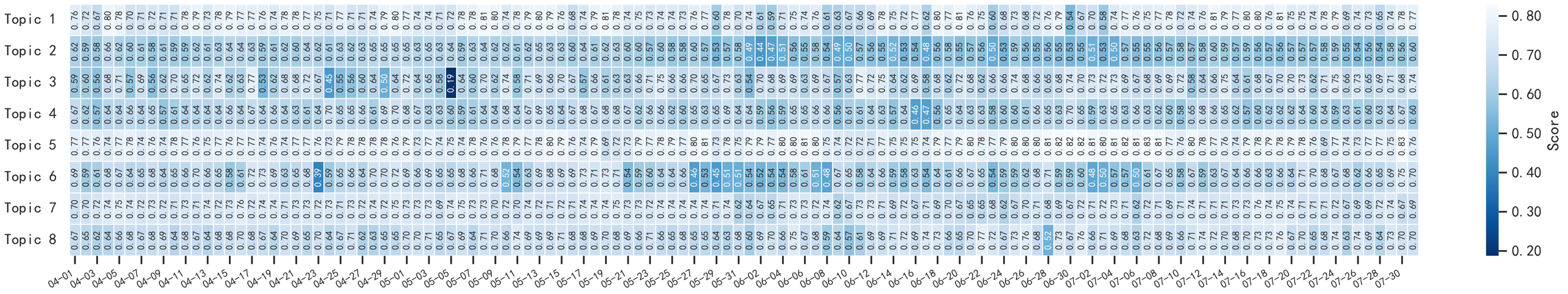

Supplement: Multimedia Appendix 2 [file jmir_v26i1e58518_app2.pdf]
